# A single-center experience of over 600 cases of robotic cholecystectomy: a propensity score match analysis

**DOI:** 10.1007/s00464-025-12454-1

**Published:** 2025-12-08

**Authors:** Francesco Celotto, Niccolò Ramacciotti, Giacomo Danieli, Gaya Spolverato, Luca Morelli, Francesco Maria Bianco

**Affiliations:** 1 Department of Surgical, Oncological and Gastroenterological Sciences, University of Padova, Padova, Italy; 2 Division of General, Minimally Invasive, and Robotic Surgery, Department of Surgery, University of Illinois at Chicago, Chicago, IL USA; 3 Department of Translational Research and New Technologies in Medicine and Surgery, University of Pisa, Pisa, Italy

**Keywords:** Single-Port, Robotic surgery, Cholecystectomy, Mininvasive surgery

## Abstract

**Background:**

Minimally invasive surgery is the gold standard for cholecystectomy and robotic-assisted cholecystectomy has been shown to be comparable if not better than the laparoscopic technique, in terms of postoperative outcomes.

We aim to compare outcomes of different robotic approaches for cholecystectomy in a high-volume Center with a large experience in robotic surgery in using robotic multiport (MP), robotic single-site (SS) and robotic single-port approach (SP).

**Methods:**

Data from 611 patients who underwent elective robotic cholecystectomy with MP, SS or SP technique were retrospectively collected between 2012 and 2024. All surgeries were performed by the same surgeon. Urgent cholecystectomies were excluded. A propensity score weighted analysis were used to balance the three groups and short-term outcomes were compared.

**Results:**

After the weighting using general characteristics (age, gender, BMI, ASA scale, comorbidity, and previous abdominal surgery), three groups of patients were obtained: MP *n* = 128; SP *n* = 116; SS *n* = 118. The three groups were homogenous for the above-mentioned characteristics. Based on linear regression analysis, the SP group had shorter overall operative time (OT), length of stay (LOS) and lower estimated blood loss (EBL), although the latter in a non-clinically significant manner (5 mL). No differences between groups in 30-day Clavien-Dindo complications, conversion rate, 30-day reinterventions, 30-day readmission and 30-day mortality (*p* > 0.05).

**Conclusion:**

The analysis between robotic cholecystectomy methods showed that the SP technique results in lower LOS and EBL than the multiport technique. Regarding the duration of surgery, the SP technique was faster than the other two methods compared.

SP robotic cholecystectomy is a safe and feasible alternative to multiport and single-site robotic surgery with promising results.

Gallstone disease is a prevalent condition in developed countries, affecting approximately 10 to 15% of the adult population. About one-fifth of these cases lead to symptoms requiring surgical intervention, making cholecystectomy one of the most frequently performed procedures in general surgery today [1]. Since its introduction in 1985 laparoscopic cholecystectomy has become the gold standard for gallbladder removal [2]. Laparoscopic cholecystectomy is recognized as the primary approach, and it is one of the main performed operations in general surgery with over 750,000 operations in the United States annually [3].

However, with the advent of new technologies and the aim of implementing new, less invasive techniques, several options for the treatment of gallbladder disease have emerged, including single-incision laparoscopic cholecystectomy (SILS), robotic-assisted laparoscopic cholecystectomy, single-site robotic cholecystectomy and single-port robotic cholecystectomy [4–6].

In the group of minimally invasive surgical techniques, SILS presented as an innovation meant to reduce invasiveness and surgical trauma. SILS has emerged as a valid alternative in various surgical procedures offering benefits, such as better short‐term and long‐term cosmesis and body image, reduced postoperative pain, quicker recovery, and improved aesthetic outcomes, compared to traditional multiport laparoscopy [7]. However, its widespread adoption has been impeded by technical challenges like limited maneuverability, instrument interference, and ergonomic issues [8]. To address these limitations, dedicated instrumentations were developed for the Da Vinci surgical systems Si and Xi (Da Vinci Single‐Site) in 2011, but its uptake was hindered by the persistence of several drawbacks, mainly the lack of endowrist, instrument excessive flexibility and insufficient strength, preventing its widespread adoption. A common concern related to the use of the single-site approach was the increased risk of incisional hernia formation [9].

The da Vinci single-port robotic platform (DVSP) is designed specifically for single-incision surgery, and has garnered interest for its potential to overcome the previous technology limitations and expand the range of surgical applications for single-site surgery. This platform presents similar functionalities of the da Vinci multiport system, featuring three wristed instruments and a 3D-HD articulating scope all deployed through a single-port. This improved technology allows for enhanced instrument maneuverability, extensive range of motion, and 360° multi-quadrant access through a single small incision of 25 mm [10].

The use of DVSP to perform cholecystectomy has been described in several papers and is a procedure performed in several centers around the world [11]. It has been described as a procedure that can be transferred easily between multiport and single-port platforms, and that has a rapid learning curve [12]. DVSP has been CE-marked in Europe for about a year now, while in the US it has FDA approval for urological and transoral surgery. In some centers, such as the University of Illinois at Chicago, DVSP is being used on an experimental basis as part of an approved protocol in general surgery and cholecystectomy is one of the most commonly performed operations.

This study aims to evaluate the short-term outcomes of robotic cholecystectomy, comparing three techniques—multi-port, single-site, and single-port—based on data from 611 procedures performed by a single surgeon at our Institution between 2012 and 2024.

## Materials and methods

### Study design and population

This study was conducted under an Institutional Review Board (IRB) approved protocol (IRB #2021‐0520). This study was written following the standards of the STROBE guidelines for observational studies [13].

The data from 611 patients who underwent robotic cholecystectomy from 2012 to 2024 were retrospectively analyzed using a prospectively collected database. All surgeries were performed at the University of Illinois at Chicago by a single surgeon (FMB) with experience in minimally invasive laparoscopic and robotic surgical techniques. The data were divided into three categories based on the robotic approach chosen: multiport robotic cholecystectomy, single-site robotic cholecystectomy, single-port robotic cholecystectomy. At our institution when performing robotic cholecystectomies with the multi-port robotic approach, four incisions of approximately 8 mm each were created to accommodate the robotic trocars, one of which was subsequently enlarged to allow for the removal of the surgical specimen. In contrast, in robotic cholecystectomies with the single-site or single-port approach, a single fascial incision of approximately 30 and 25 mm, respectively, was created to accommodate the robotic port. In the three groups the fascial incision was repaired by direct suture with either a 0 Polyglactin 910 suture or 1 Polydioxanone. Case selection was performed for the SS and SP techniques, excluding morbidly obese patients and patients with a history of abdominal surgery for the first 10 operations.

In this analysis, cholecystectomies indicated for acute cholecystitis were excluded from all groups. Indications for elective cholecystectomy included symptomatic cholelithiasis, porcelain gallbladder, and chronic cholecystitis, gallbladder polyps, gallstone pancreatitis and choledocholithiasis. When choledocholithiasis was suspected, patients underwent magnetic resonance cholangiography (MRC) prior to surgery, followed by endoscopic retrograde cholangiopancreatography (ERCP) if indicated for the removal of bile duct stones.

Intraoperative cholangiography was performed in cases of suspected or intraoperatively confirmed choledocholithiasis that had not been identified in preparatory examinations, regardless of the robotic platform used.

### Variables and outcomes

Perioperative variables were collected using standardized case report forms and entered prospectively into an institutional database. Data collected included age, gender, body mass index (BMI), American Society of Anesthesiologists (ASA) classification, presence of comorbidities, previous abdominal surgeries. Perioperative variables such as overall time (OT), Estimated Blood Loss (EBL), conversion (to laparoscopy or to open surgery) were also registered.

Recovery parameters such as recovery time, length of hospital stay (LOS), overall 30-day complication, 30-day reintervention, 30-day readmission, mortality were also considered for analysis. The long-term outcome was defined as the occurrence of an incisional hernia after the index surgery.

The primary outcome was to evaluate the quantification of intra-operative and early post-operative outcomes. Follow-up evaluation was scheduled in the office two weeks after surgery, with additional telephone consultations at different intervals. If telephone screenings revealed any positive findings, patients were then scheduled for in-person physical examinations at the office.

### Analysis

Propensity score matching (PSM) was implemented to mitigate selection bias caused by disparities in sample sizes and other covariates. To assess the effect of treatment on the treated subjects (ATT—Average Treatment Effect on the Treated), we employed a weighting method based on logistic regression to balance covariates across the treatment groups. The features included in PSM were, age, sex, body mass index (BMI), American Society of Anaesthesiologists (ASA) physical status classification system, presence of comorbidities and previous surgeries. After the PSM, the covariates exhibit a sufficiently small residual difference (< 0.1), indicating good balance. The groups were composed of 128, 116 and 118 patients, respectively, for the MP, SS and SP group. The results were compared using the Wilcoxon rank-sum test for complex survey samples and the chi-squared test with Rao & Scott’s second-order correction.

### Ethics approval and consents

This study was conducted under an Institutional Review Board (IRB) approved protocol (IRB #2021‐0520) by the University of Illinois at Chicago. Patients who underwent SPRC were provided with comprehensive explanations regarding the procedure and its methodology, explaining both expected outcomes and potential risks. Additionally, prior to obtaining written consent, patients were educated on other viable approaches, including laparoscopic and other robotic techniques.

## Results

The patients that underwent elective surgery were divided into three group based on the approach chosen for the procedure, 129 patients for the multiport approach (MP), 242 for the single-site approach (SS) and 240 for the single-port approach (SP); details of the patients can be found in Table 1.

**Table 1:** Patients’ characteristics

Characteristic	Multiport (129)	Single site (242)	Single port (240)
Age	47 (18–91)	40 (14–88)	42 (18–76)
*Sex*			
Male	37	46	41
Female	92	196	199
BMI	34 (18–63)	32 (17–57)	34 (15–72)
ASA			
1	14	59	26
2	73	143	140
3	39	40	72
4	3	-	4
Comorbidities			
No	103	172	123
Yes	26	172	117
Previous surgery			
No	63	108	91
Yes	66	134	149
Indication			
Symptomatic cholelithiasis	111	207	220
Biliary dyskinesia	2	2	1
Gallbladder polyps	3	7	7
Chronic cholecystitis	12	25	8
Choledocal cyst	1	-	-
Porcelain gallbladder	-	1	4

*BMI* body mass index, *ASA* American Society of Anaesthesiologists physical status classification system

The groups were analyzed after propensity score matching. Three hundred and sixty-two patients were identified, *n* = 128 in the MP group, *n* = 116 in the SS group and n = 118 in the SP group.

**Fig. 1:**
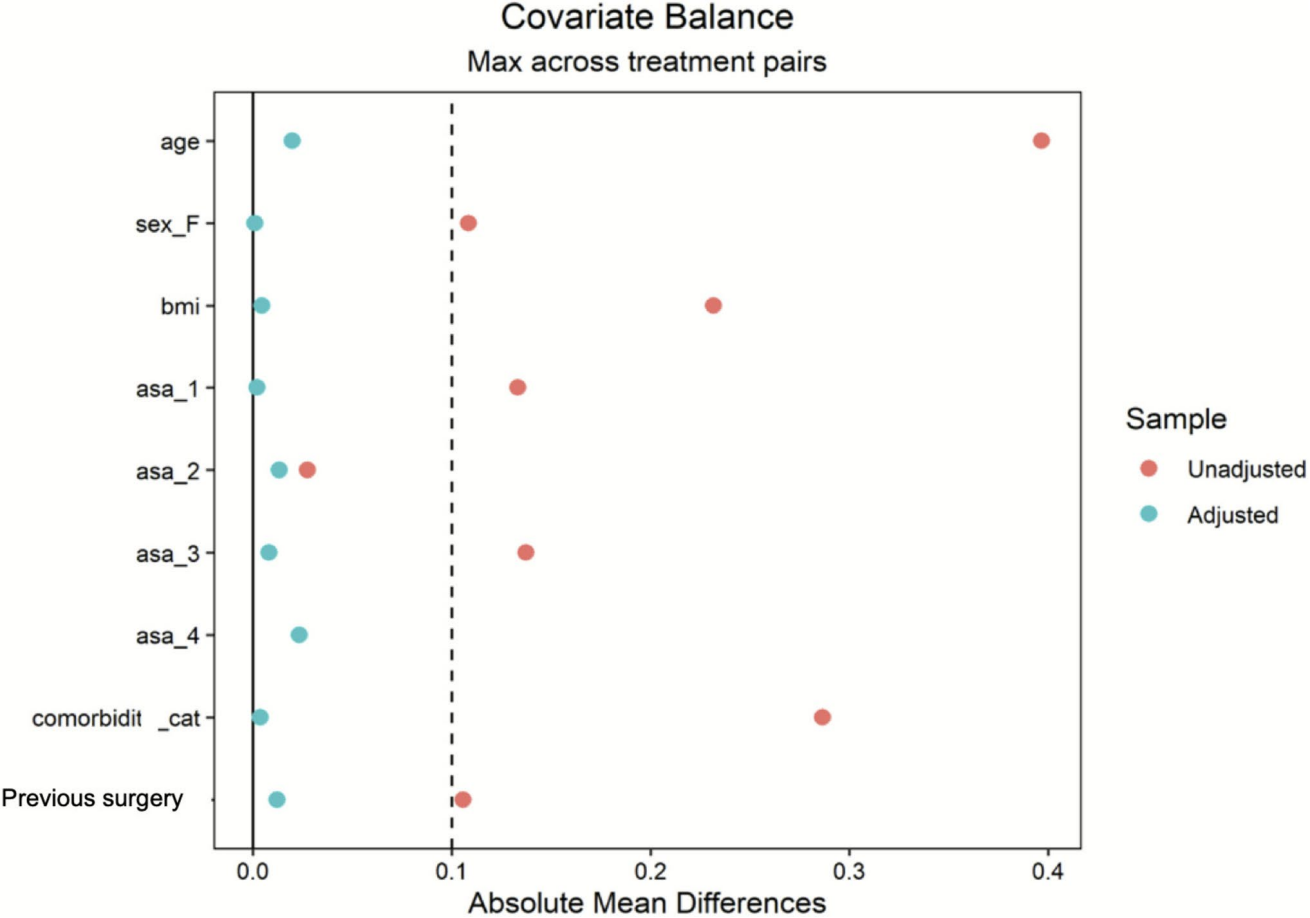
Plot of covariates before (red dots) and after adjustment (green dots). *BMI* Body mass index, *ASA* American Society of Anaesthesiologists physical status classification system

As shown in Fig. 1, the groups are particularly homogeneous after matching.

The general characteristics of the 362 included patients are shown in Table 2.

**Table 2:** Patients’ characteristics after propensity score matching

Characteristic	Multiport (128)	Single site (116)	Single port (118)	p-value
Age	45 (32, 59)	48 (36, 55)	46 (32, 58)	> 0.9
Sex				0.8
Male	36 (28%)	31 (26%)	28 (24%)	
Female	92 (72%)	88 (74%)	88 (76%)	
BMI	33 (28, 39)	33 (27, 39)	33 (28, 39)	> 0.9
ASA				0.5
1	14 (11%)	14 (12%)	12 (11%)	
2	72 (56%)	71 (60%)	64 (55%)	
3	39 (30%)	32 (27%)	38 (33%)	
4	3 (2.3%)	0 (0%)	1 (0.9%)	
Comorbidities				0.8
No	25 (20%)	25 (21%)	26 (22%)	
Yes	103 (80%)	93 (79%)	90 (78%)	
Previous surgery				> 0.9
No	65 (51%)	63 (53%)	61 (52%)	
Yes	63 (49%)	55 (47%)	55 (48%)	
Indication				0.8
Symptomatic cholelithiasis	111	103	104	
Biliary dyskinesia	2	2	1	
Gallbladder polyps	2	3	3	
Chronic cholecystitis	12	8	8	
Choledocal cyst	1	-	-	

*EBL* estimate blood loss, *LOS* length of stay, *BMI* body mass index, *ASA* American Society of Anaesthesiologists physical status classification system

Intraoperative data as well as post-operative morbidity and mortality were recorded and were compared among the three groups; these data are shown in Table 3.

**Table 3:** Short term outcomes

Characteristic	Multiport (128)	Single site (118)	Single port (116)	p-value
Overall time (min)	80 (64, 100)	80 (67, 95)	56 (46, 64)	< 0.001
EBL (mL)	10 (5, 20)	10 (5, 15)	5 (5, 5)	< 0.001
LOS (days)	1.37 (1, 42)	0.24 (0, 9)	0.16 (0, 4)	< 0.001
Conversion (%)				0.2
No	127 (99)	127 (99)	116 (100)	
Yes (open)	1 (0.8)	1 (0.8)	0 (0)	
Yes (robotic multiport)	0 (0)	0 (0)	0 (0)	
Yes (laparoscopic	0 (0)	0 (0)	0 (0	
Clavien-Dindo complications (%)				0.6
1-3a	8 (6.3)	9 (7.6)	4 (3.4)	
3b-4	2 (1.5)	1 (0.8)	0 (0	
Bile leak	0 (0)	1 (0.8)	1 (0.8)	0.6
Bile duct injury	0 (0)	0 (0)	0 (0)	1
30-day reoperation (%)	0 (0)	0 (0)	0 (0)	1
30-day readmission (%)	3 (2.3)	3 (2.5)	2 (1.7)	0.9
30-day mortality (%)	0 (0)	0 (0)	0 (0)	1
Incisional hernia occurrence (%)	1 (0,8)	4 (3,1)	2 (1,7)	0.33

*EBL* estimate blood loss, *LOS* length of stay

Overall Time (OT) is defined as the time between skin incision and skin closure. The OT in the SP group was found to be less than the MP and SS group (*p* < 0.001), with an average of 56 min (46–64). OT in the MP group was 80 min (64–100), and average OT in the SS group was 80 min (67–95).

EBL was statistically lower in the SP group than in the MP and SS groups (p < 0.001), although this difference was not clinically significant. The median EBL in the SP group was 5 mL (1–5 mL), compared to 10 mL (5–20) in the MP, and 10 mL (5–15) in the SS groups.

The average LOS in the MP group was 1,37 (1–42) days while average LOS in the SS group was 0,24 (0–9) days, and in the SP group was 0,16 (0–4) days. LOS in SP and SS groups was found to be less than the MP group (p < 0.001).

There were no differences between the methods for conversions, 1 (0.8%) occurred in the MP group, 5 (4.2%) in the SS group, 3 (2.5%) of which were converted to a multiport robotic technique and 2 (1.7%) to a multiport laparoscopic technique, and 0 in the SP group (*p* = 0.4). There was no difference in Clavien-Dindo complications between the groups (*p *= 0.6). In particular, there was no difference in the incidence of bile leaks (*p* = 0.6) or bile duct injuries, which did not occur in any group. There were no re-interventions or fatalities in any of the groups within 30 days of surgery.

There were no differences between the groups (*p* = 0.9) in terms of 30-day readmission either (MP 3 (2.3%); SS 3 (2.5%); SP 2 (1.7%)).

In the long-term, the incisional hernia rate was found to be 1 (0.8%) in the MP group, 4 (3.1%) in the SS group and 2 (1.7%) in the SP group. The median follow-up period was 8 months in the MP group, 10.3 months in the SS group and 13.5 months in the SP group. There were no differences between the three groups (*p* = 0.33).

## Discussion

Minimally invasive surgery (MIS) has advanced beyond traditional laparoscopic techniques, ushering in a new era of robotic-assisted procedures. Robotic surgery addresses many of the ergonomic and technical challenges inherent to laparoscopy, enabling surgeons to perform complex operations with enhanced precision and control. Since their introduction in the late 1990s, robotic systems have undergone continuous innovation, culminating in the development of single-port (SP) robotic platforms. These systems, designed to operate through a single small incision, represent the next frontier in MIS, combining reduced invasiveness with improved maneuverability and possibly surgical outcomes [4]. Between the advent of multi-port robotic surgery and robotic surgery with dedicated single-port robots, there was a transitional period with the use of a single-incision surgical system proposed by Intuitive in 2011 [14]. Although this technology improved some of the technical limitations of traditional SILS, the excessive flexibility of the instrument, the lack of endowrist and the limited strenght prevented its widespread adoption.

At our institution, cholecystectomy has been performed with the da Vinci Si/Xi multiport robotic system since 2012, which was associated with the use of the Intuitive single-incision multiport robotic instrumentation in the Si system. From 2019, with the introduction of the DVSP, used under IRB, single-incision multiport robotic procedures will be replaced by the DVSP.

The comparison of the robotic methods (MP, SS, SP) of performing cholecystectomy presented in this study allows several considerations to be made about the results obtained and the short-term outcomes.

Multiport robotic cholecystectomy, despite several advantages over the traditional laparoscopic procedure, such as lesser duration of stay and lesser readmission rate within 90 days of the index operation, easier use of intraoperative ICG-fluorescence imaging, reduced post-operative pain and conversion rate in acute setting, has not overcome the laparoscopic cholecystectomy yet due to the longer operative time and hospital cost [3, 15].

In a recent meta-analysis published by Delgado LM et al.[16] based on data from 22,440 patients who underwent laparoscopic or robotic multiport cholecystectomy, they found that robotic cholecystectomy is associated with a longer total time than laparoscopic cholecystectomy. In our case series the SP approach demonstrated significantly shorter operative times compared to both MP and SS methods, with an average of 56 min versus 80 min for the other two techniques. The OT of SP cholecystectomy reported in our work is similar to the OT of laparoscopic cholecystectomy reported in several papers included in the meta-analysis by Delgado LM et al.[17, 18]. Regarding the difference between SP and SS several papers in the literature are consistent with the results of the present case series. Choi YJ et al.[19] in a case series of single-incision cholecystectomies, of which 118 Si/Xi vs. 216 SP, report a significantly faster total operative time in the SP group. Similarly, Cruz CJ et al.[11] report a lower OT of over 30 min with the SP technique compared to SS. This result is likely attributed to the streamlined nature of the SP system, which eliminates the need for multiple port placements and optimizes instrumentation maneuverability. The SP technique was associated with the shortest LOS (0.16 days), followed closely by the SS group (0.24 days). In contrast, the MP group had a considerably longer LOS (1.37 days), although it is still in line with, if not inferior to, the data available in the literature. In our case series of SS and SP cholecystectomy, the LOS is lower than in other case series reported in the literature. Cruz CJ et al.[11] report a LOS of 1.9 ± 1.0 days for SS and 1.5 ± 0.7 days for SP. Choi YJ et al.[19] report a LOS for SS of 2.43 ± 0.947 days and for SP of 2.39 ± 0.71 days. The shorter duration of LOS for SP and SS may be a consequence of less invasiveness, reduced postoperative pain and faster recovery time. A significant reduction in recovery time was reported by Dreifuss N et al.[20] even in day surgery applications of DVSP like inguinal hernia compared to multiport inguinal hernias.

Regarding EBL, while the SP group had statistically lower EBL compared to MP and SS groups, the differences were not clinically significant (median 5 mL vs. 10 mL). This finding suggests that although SP has technical advantages, in our series all three techniques are safe in terms of intraoperative bleeding.

There was no difference in Clavien-Dindo complications between the groups. Both mild to moderate (I-IIIa) and severe (IIIb-IV) complications were less frequent in the SP group, although this difference was not significant (*p* = 0.565). Furthermore, when analyzing the specific and most feared complications of cholecystectomy, bile leak and bile duct injury, no difference was found between the groups. No bile leaks occurred in the MP group, whereas 1 (0.8%) occurred in both the SS and SP groups (*p* = 0.577). In both cases it was successfully treated with endoscopic Stent placement. No major bile duct injury was observed in any of the three groups, whereas incidences of major bile duct injury vary between 0.08 and 0.3% in laparoscopic cholecystectomy. A recent study by Kalata et al.[21], which retrospectively analyzed Medicare data from 1,026,088 patients, found that robotic cholecystectomy was associated with a higher incidence of bile duct injuries requiring definitive surgical repair within one year compared to laparoscopic cholecystectomy (LC). These findings were challenged in the meta-analysis by Delgado LM et al.[16], which included only data from randomized clinical trials and observational studies with propensity score matching, encompassing over 22,000 patients. No differences were found in the incidence of complications, including bile leaks or bile duct injuries, between robotic and laparoscopic cholecystectomy. In our case series, the incidence of bile leak and bile duct injury is lower in all three groups than in the much larger case series reported in the above studies. No significant differences were observed in conversion rates, reoperations, or mortality across the groups.

This is the first study to analyze the short-term outcomes of robotic cholecystectomy using the three techniques currently available. After propensity score matching between groups, the SP approach was shown to have advantages over multiport and single-site multiport. However, there are some questions and limitations that have to be addressed.

One of the unanswered questions about robotic surgery is the high cost of surgery. Using the SP robotic platform increases the cost of a cholecystectomy by approximately 30% compared with the MP technique. This reflects the natural trend in technological development, in which newer platforms and dedicated instruments carry higher acquisition and maintenance expenses. The financial impact of these differences cannot be understated, and it could represent a limitation to the widespread applicability of SP surgery.

At our institution, however, the majority of patients in all three groups (MP, SS, and SP) were scheduled and treated safely in an outpatient setting. Based on our experience and the results of this study, we can state that SP cholecystectomy can be reliably performed on an outpatient basis. The procedure takes a similar amount of time to laparoscopy, and patients can be discharged from hospital quickly without compromising safety. This organizational aspect has practical implications: the combination of shorter operative times and near-universal outpatient feasibility may allow a higher number of procedures to be performed within the same operative block, potentially increasing procedural throughput and, consequently, institutional revenue. While this represents a plausible mechanism through which higher per-case costs might be partially offset, we acknowledge that this interpretation remains speculative in the absence of dedicated economic analyses.

Previous studies indicate that in high-volume centers, the cost differential between laparoscopic and robotic procedures can be mitigated thanks to decreased hospital length of stay and this expenditure appears to be justifiable in high-volume centers where it can be amortized over a larger number of cases [22].

Our case series of robotic SP cholecystectomies shows that DVSP allows shorter operative times compared to other robotic methods, comparable to laparoscopic cases in the literature [23]. Furthermore, the average length of stay in the SP group was 124.75 min. Combined with the short duration of the surgery, this result supports the possibility of performing robotic SP cholecystectomies on an outpatient basis, thereby increasing efficiency and patient turnover.

Another issue related to the use of SP is the open question of the risk of incisional hernia formation due to the larger incision size for insertion of the 2.5 cm port. This was already a concern with the SS technique. In the long-term, the incisional hernia rate was found to be 1 (0.8%) in the MP group, 4 (3.1%) in the SS group and 2 (1.7%) in the SP group. In a meta-analysis by Jensen S et al. [6] the weighted average incidence rate of incisional hernia at 12 months after SS cholecystectomy was 4.5%. Regarding SP, Bianco et al. reported a comparable incisional hernia rate after 13 months of follow-up between multi-port (1.1%) and single-port (2.3%) robotic TAPP [8]. Future analysis of long-term outcomes, including follow-up for incisional hernia at the port site, is mandatory.

The retrospective design of the study, combined with its relatively small sample size, may limit the generalizability of the findings. Additionally, the research was conducted at a single high-volume center by an experienced surgeon with a strong background in both laparoscopic and robotic surgery. While this enhances the robustness of the results, it may not fully account for the learning curve of surgeons with varying levels of expertise, limiting the applicability of the findings to other settings or less experienced practitioners. A single surgeon’s reported experience may introduce bias, as it may reflect a reduction in operating times linked to the surgeon’s own progressive experience. However, it has been observed that console operating time does not vary during the learning curve of DVSP cholecystectomy [12]. Consequently, the lower OT observed in the SP group compared to the SS and MP groups is believed to be justified by faster preparation and docking times for the platform.

The findings reported in this paper suggest that the SP robotic platform may improve surgical outcomes in robotic single-access cholecystectomy by providing enhanced ergonomics and refined instrument maneuverability. By performing all functions through a single-incision, the SP system reduces invasiveness while maintaining safety, and could be considered a technically feasible alternative to elective robotic cholecystectomy, despite being more expensive. Further studies are warranted to confirm these results in multi-institutional settings, evaluate long-term outcomes, and assess cost-effectiveness to determine the broader applicability of the SP approach.

The introduction of the SP platform at our center reflects a conscious strategic investment driven by an institutional commitment to technological advancement. As a high-volume academic program with extensive experience in robotic surgery, our institution views the adoption of emerging technologies not only to improve clinical outcomes and patient experience, but also as an integral part of its mission in innovation and surgical education, even when this implies higher upfront or per-case expenditures. Future multicenter cost-effectiveness analyses will be essential to determine whether the increased efficiency observed with SP, such as reduced length of stay, outpatient management, and faster operative workflow, translates into meaningful economic benefits for healthcare systems.

## Conclusions

The introduction of the single-port robotic platform could contribute to the development of robotic cholecystectomy further, potentially reducing surgical trauma and operating time. However, the single-port platform is more expensive than the multi-port platform. In our analysis comparing robotic multiport, single-site, and single-port cholecystectomies, the DVSP platform demonstrated shorter operative times and encouraging short-term clinical outcomes. Additional studies are needed to evaluate long-term results, particularly regarding the potential occurrence of incisional hernias, as well as to assess the economic sustainability of this approach for routine clinical use.
